# Nrf2 signaling in chronic obstructive pulmonary disease: regulation of ferroptosis and therapeutic implications

**DOI:** 10.1016/j.redox.2025.103931

**Published:** 2025-11-14

**Authors:** Qian Gao, Yayun Mao, Shu Xie, Dandan Liu, Yifan Lv, Xiaodan Liu, Weibing wu

**Affiliations:** a School of Exercise and Health, Shanghai University of Sport, Shanghai, China; b School of Rehabilitation Science, Shanghai University of Traditional Chinese Medicine, Shanghai, China

**Keywords:** Chronic obstructive pulmonary disease, Nrf2, Ferroptosis, Oxidative stress, Lipid peroxidation

## Abstract

Chronic obstructive pulmonary disease (COPD), a major global cause of morbidity and mortality, is pathologically defined by persistent oxidative stress, chronic inflammation, and dysregulated cell death pathways. This review critically examines the emerging role of the transcription factor Nrf2 in modulating these processes within COPD, with particular emphasis on its potential regulation of ferroptosis, a novel iron-dependent form of regulated cell death implicated in disease pathogenesis. We explore the complex regulation of Nrf2, including both Keap1-dependent and -independent degradation mechanisms, as well as its upstream activators and downstream effectors in the context of COPD. Notably, we provide evidence that Nrf2 dysfunction weakens cellular defenses against oxidative stress and inflammation while increasing susceptibility to ferroptosis. Ferroptosis, marked by iron accumulation, glutathione (GSH) depletion, glutathione peroxidase 4 (GPX4) inactivation, and lipid peroxidation, is emerging as a key driver of airway epithelial damage, emphysema, and inflammation in COPD. We also delve into the molecular mechanisms of the Nrf2-ferroptosis axis, highlighting the role of Nrf2 in regulating iron homeostasis, the System Xc^−^/GSH/GPX4 pathway, and lipid peroxidation, with additional crosstalk to pathways like peroxisome proliferator-activated receptor gamma (PPARγ). These insights underscore the potential for targeting Nrf2-mediated ferroptosis in developing novel therapeutic approaches to combat COPD.

## Graphical abstract

**Figure:**
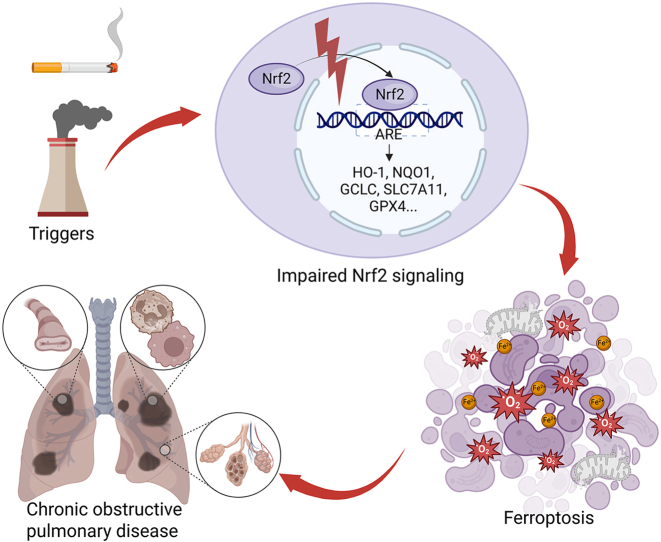

## 1 Introduction

Chronic obstructive pulmonary disease (COPD) imposes a substantial and growing global health burden, ranking among the top three causes of death worldwide. Pathologically, COPD is characterized by persistent oxidative stress, chronic airway and parenchymal inflammation, and aberrant cell death mechanisms [1]. These interconnected processes drive the progressive airflow limitation and structural damage central to the disease. A key driver underlying this pathology is the impairment of endogenous antioxidant defenses, with dysregulation of the nuclear factor erythroid 2-related factor 2 (Nrf2) pathway representing a pivotal mechanism [2,3].

Nrf2 is a master transcriptional regulator of cellular redox homeostasis, orchestrating the expression of a wide range of cytoprotective genes encoding antioxidant enzymes, phase II detoxifying enzymes, and anti-inflammatory mediators [4,5]. Under physiological conditions, Nrf2 activity is tightly regulated by mechanisms such as Kelch-like ECH-associated protein 1 (Keap1)-dependent degradation, β-transducing repeat-containing protein (βTrCP)-mediated degradation, and HMG-CoA reductase degradation protein 1 (HRD1) or WDR23/Cullin (Cul)4-dependent pathways [[6], [7], [8], [9]]. Activation of Nrf2, triggered by electrophiles or reactive oxygen species (ROS), leads to its dissociation from Keap1, nuclear translocation, heterodimerization with small Maf (sMaf) proteins, and binding to antioxidant response elements (AREs) [10]. This results in the induction of genes such as heme oxygenase-1 (HO-1), NAD(P)H quinone oxidoreductase 1 (NQO1), glutamate-cysteine ligase catalytic subunit (GCLC), and solute carrier family 7 member 11 (SLC7A11).

Impaired Nrf2 signaling has been implicated in the pathogenesis of COPD. Reduced Nrf2 expression and activity, observed in both clinical samples and experimental models, compromise cellular defenses against oxidative stress and inflammation, contributing to disease progression [11,12]. Emerging research also highlights a critical link between Nrf2 dysfunction and ferroptosis, a novel form of regulated cell death [13,14]. Ferroptosis is characterized by iron-dependent lipid peroxidation, driven by dysregulated iron metabolism and glutathione peroxidase (GPX)4 inactivation with glutathione (GSH) depletion [15,16]. This process is increasingly recognized as a key driver of airway epithelial damage, emphysema, and inflammation in COPD [17,18]. The Nrf2 pathway plays a multifaceted role in regulating ferroptosis susceptibility by controlling iron homeostasis, the System Xc-/GSH/GPX4 axis, lipid peroxidation, and crosstalk with other pathways such as peroxisome proliferator-activated receptor gamma (PPARγ) [15,19].

Critically, the role of Nrf2 in COPD is distinct from its function in other pathologies. Unlike its transient activation in acute injury or constitutive activation in certain cancers, Nrf2 signaling in COPD is defined by a progressive and chronic impairment [2,20,21]. This pathognomonic feature is primarily driven by persistent environmental insults like cigarette smoke (CS), which orchestrates a dual assault on the Nrf2 pathway. It not only generates overwhelming oxidative stress that saturates cellular defenses but also directly disrupts Nrf2 expression and function through mechanisms such as promoter hypermethylation [[22], [23], [24]]. Furthermore, CS concurrently dysregulates the upstream signaling networks that normally govern Nrf2 activation, creating a scenario where the pathway is both intrinsically damaged and extrinsically suppressed [[25], [26], [27]]. The collective failure leads to a sustained defensive inadequacy. The resultant collapse of this central antioxidant node creates a unique vulnerability, permitting the unchecked propagation of oxidative stress, chronic inflammation, and, as a pivotal consequence, the escalation of ferroptosis [13,28,29].

Therefore, this review will critically dissect the unique Nrf2 pathology in COPD, moving beyond a general description of its signaling network. We will place special emphasis on its groundbreaking interplay with ferroptosis, thereby elucidating the disease-specific mechanistic rationale for targeting this axis and highlighting novel therapeutic paradigms.

## 2 The structure, regulation, and regulatory network of Nrf2 in COPD

### 2.1 The structure of Nrf2

**Fig. 1:**
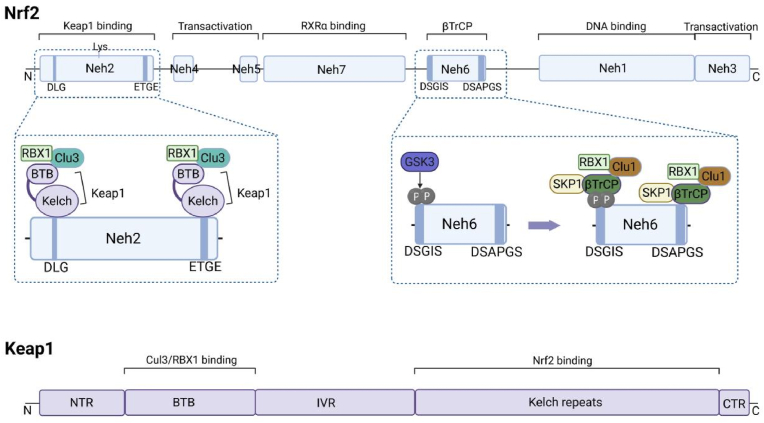
The structure of Nrf2. Nrf2 contains 605 amino acids and is organized into 7 conserved functional Nrf2-ECH homologous (Neh) domains. Keap1 consists of 5 domains.

Nrf2, encoded by the Nfe2L2 gene, is a central transcription factor within the Cap'n'Collar basic leucine zipper (CNC-bZIP) family. Comprising 605 amino acids organized into seven conserved functional Nrf2-ECH homology (Neh) domains, Nrf2 orchestrates the cellular defense against oxidative stress, inflammation, metabolic disorder, and cell death by regulating cytoprotective gene expression [30,31] (Fig. 1). The CNC-bZIP structure in the Neh1 facilitates heterodimerization with sMaf proteins to bind ARE and activate transcription [32]. Neh2 contains two conserved Keap1-binding motifs, ETGE and DLG, which interact with the Kelch domain of Keap1 in a Hinge-Latch manner [33]. Additionally, lysine residues within Neh2 serve as ubiquitin attachment sites, mediating Keap1-dependent Nrf2 degradation [34]. Neh3, Neh4, and Neh5 constitute transactivation domain of Nrf2 [35]. Neh4 and Neh5 synergistically recruit CREB-binding protein (CBP), promoting Nrf2 acetylation and transcriptional activation [36]. Furthermore, these domains interact with the E3 ubiquitin ligase HRD1, facilitating Nrf2 ubiquitination and degradation [37]. Neh6 contains conserved DSGIS and DSAPGS motifs that serve as phosphodegrons. Under basal conditions, phosphorylation of these motifs by glycogen synthase kinase-3 (GSK3) enables their binding to βTrCP, leading to Cul1/βTrCP-dependent ubiquitination and subsequent degradation of Nrf2 [38]. Neh7 functions as a regulatory module by binding retinoid X receptor alpha (RXRα). This interaction represses Nrf2 transcriptional activity by blocking co-activator access to the transactivation domains Neh4 and Neh5 [39].

### 2.2 The regulation of Nrf2

**Fig. 2:**
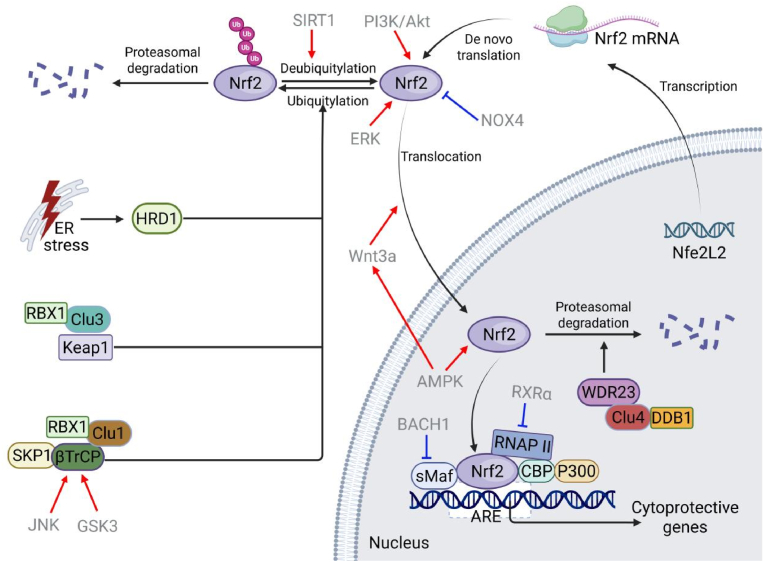
The regulation of Nrf2. Nrf2 is primarily regulated by four E3 ubiquitin ligases, including Keap1/Cul3/RBX1, βTrCP/SKP1/Cul1/RBX1, HRD1, and WDR23/Cul4/DDB1. Additionally, Nrf2 activity is regulated by various signaling pathways and cellular sensors, such as Wnta/β-catenin, PI3K/Akt, AMPK, MAPK, NOX4, and SIRT1.

The transcriptional activity of Nrf2 is primarily governed by its protein stability and subcellular localization. Under basal conditions, Nrf2 undergoes continuously degraded via the ubiquitin-proteasome system, preventing its nuclear accumulation and limiting the expression of cytoprotective genes. This rapid turnover ensures precise regulation of antioxidant and detoxification pathways. Several E3 ubiquitin ligase complexes contribute to Nrf2 stability, with the ubiquitin-proteasome system being the dominant pathway for its post-translational regulation (Fig. 2).

#### 2.2.1 Ubiquitin-proteasome system

Nrf2 stability is primarily controlled by four E3 ubiquitin ligases, including Keap1/Cul3/RING-box protein (RBX)1, βTrCP/S-phase kinase-associated protein-1 (SKP1)/Cul1/RBX1, HRD1 (also known as synoviolin or SYVN1), and WDR23/Cul4/DNA damage-binding protein 1 (DDB1).

Keap1, a cytoplasmic adaptor for the Cul3-based E3 ubiquitin ligase complex, is the primary regulator of Nrf2. Its structure comprises several domains, including amino terminal region (NTR), broad complex, tramtrack, bric-a-brac (BTB), intervening region (IVR), Kelch repeats, and *C*-terminal region (CTR) [6] (Fig. 1). Functioning as a homodimer via its BTB domain, Keap1 recruits Cul3 to form the active Keap1/Cul3/RBX1 complex that targets Nrf2 for proteasomal degradation [6]. The Kelch repeat domain binds directly to the ETGE and DLG motifs within the Neh2 domain of Nrf2 [34]. Crucially, Keap1 serves as a cellular redox sensor. Humans possess 27 redox-sensitive cysteine residues in Keap1, with specific residues in the IVR and BTB domains like C151, C226, C273, and C278, demonstrating high reactivity towards ROS and electrophiles [40,41]. Oxidation or covalent modification of these residues constitutes a “cysteine code”, which disrupts Keap1's conformation and/or its interaction with Cul3, thereby impairing ubiquitin ligase activity. Consequently, upon modification, Keap1 releases Nrf2, facilitating its nuclear translocation.

The βTrCP/SKP1/Cul1/RBX1 complex also regulates Nrf2 stability. β-TrCP, functioning as a dimer, directly binds to two conserved phosphodegron motifs within the Neh6 domain: DSGIS and DSAPGS in humans, utilizing its WD40 repeat domains [7]. Phosphorylation of the *C*-terminal serine residue within the DSGIS motif by GSK3, particularly GSK3β, is crucial for high-affinity βTrCP binding. In contrast, recognition of the DSAPGS motif occurs independently of GSK3 activity [7,38]. Following GSK3-mediated phosphorylation, the βTrCP/SKP1/Cul1/RBX1 complex polyubiquitinates Nrf2, primarily targeting lysine residues adjacent to the Neh6 domain, as Neh6 itself lacks lysines, marking it for proteasomal degradation [7,42]. A distinct degradation pathway involves HRD1, an endoplasmic reticulum (ER) membrane-associated E3 ubiquitin ligase and a component of the ER-associated degradation machinery. Upregulated during ER stress, HRD1 interacts directly and specifically with the Neh4 and Neh5 transactivation domains of Nrf2 in an adaptor-independent manner [9]. WD40-repeat protein WDR23 functions as a substrate-specific adaptor for the Cul4/DDB1 E3 ubiquitin ligase complex. WDR23 recognizes a conserved amino acid sequence motif near the DLG motif within Neh2 domain of Nrf2, thereby bridging Nrf2 to the Cul4/DDB1 core complex [8]. This interaction enables WDR23/Cul4/DDB1-mediated ubiquitination of Nrf2 in a Keap1-independent manner, targeting it for proteasomal degradation.

#### 2.2.2 Proteasome-independent regulation

Kinase-mediated phosphorylation influences Nrf2 stability and activity. Phosphorylation by kinases such as PI3K/Akt, *c*-Jun *N*-terminal kinase (JNK), extracellular signal-regulated kinases (ERK), casein kinase 2, protein kinase C (PKC), and protein kinase R (PKR)-like endoplasmic reticulum kinase (PERK) generally promotes Nrf2 accumulation and transcriptional activation. Conversely, phosphorylation by p38 and GSK-3β destabilizes Nrf2 [43].

Nuclear proteins also regulate Nrf2 through modulation of its acetylation status. The association of RXRα with Nrf2 antagonizes the binding of transcriptional co-activators CBP and p300 to the Neh4 and Neh5 domains. This prevents acetylation of lysine residues within the Neh1 domain, thereby reducing Nrf2's DNA binding affinity for the ARE. Furthermore, RXRα binding to the Neh7 domain directly inhibits RNA polymerase II recruitment to ARE-containing gene promoters, leading to the downregulation of Nrf2 target genes [39]. Additionally, BTB and CNC homology 1, a CNC-bZIP repressor, competes with Nrf2 for binding to sMaf proteins, consequently repressing transcription of ARE-regulated genes [44].

### 2.3 Nrf2 regulatory network in COPD pathogenesis

The aforementioned regulatory mechanisms governing Nrf2 stability and activity are critically dysregulated in the context of COPD. A complex network of upstream signaling pathways and cellular sensors, often impaired by disease-specific etiology like CS, converges to modulate Nrf2 function, thereby contributing significantly to disease progression [11,21,[45], [46], [47]]. The major upstream regulators of Nrf2, their key molecular components, functional roles in COPD, and corresponding therapeutic agents are systematically summarized in Table 1.

**Table 1:** Major upstream pathways regulating Nrf2 and therapeutic interventions in COPD.

Regulatory pathway/sensor	Key molecules	Function in COPD	Representative therapeutic agents
Wnt/β-catenin	Wnt3a, β-catenin, GSK-3β	Promotes Nrf2 activation and nuclear translocation; attenuates emphysema and inflammation. Unidirectional regulation (Wnt to Nrf2).	Lithium chloride, metformin (via AMPK)
AMPK	AMPK, LKB1	Cellular energy sensor; its activation enhances Nrf2 signaling and suppresses inflammation.	Metformin, triterpene acids
PI3K/Akt	PI3K, Akt (PKB)	Dual role. Generally promotes Nrf2 stability and activation. CS inhibits this axis, exacerbating oxidative stress.	Bu-Shen-Fang-Chuan formula
MAPK	ERK, JNK, p38	ERK activation generally promotes Nrf2 nuclear translocation. JNK/p38 inhibition reduces oxidative stress and apoptosis.	Recuperating lung decoction (inhibits ERK), BFP-TA (inhibits JNK/p38)
Kinases	PKC, PERK, CK2	Phosphorylate Nrf2, promoting its dissociation from Keap1 and nuclear accumulation.	Crocin (implicated), various phytochemicals
SIRT1	SIRT1, NAD^+^	Deacetylates and activates Nrf2; enhances antioxidant gene expression.	Astaxanthin, BFP-TA
NOX4	NOX4, ROS	Major ROS source in COPD. Overproduction suppresses Nrf2 pathway, creating oxidative stress.	Phycocyanin (inhibits NOX2/4)
DJ-1/Wnt	DJ-1, Wnt3a, β-catenin	Stabilizes Nrf2 by activating Wnt signaling; protects against oxidative stress.	/
Emerging regulators	Pkm2, CYP2A13, TXNRD1, DAMPs	Modulate Nrf2 activity through metabolic reprogramming, xenobiotic response, and danger signaling.	Effective component of Bufei Yishen formula III (targets Pkm2)

The evolutionarily conserved Wnt3a/β-catenin pathway, involved in lung development and repair, exerts protective effects in COPD partly by activating Nrf2 [27,48]. Treatment with the Wnt pathway activator lithium chloride attenuates elastase-induced emphysema and cigarette smoke extract (CSE)-induced lung inflammation in wild-type mice. This protection correlates with enhanced Nrf2 activation and upregulation of HO-1 and NQO1 expression. Crucially, these benefits are absent in Nrf2-deficient mice, confirming Nrf2 as the key mediator of Wnt3a/β-catenin-dependent protection. In normal human bronchial epithelial (NHBE) cells, Wnt3a overexpression increases Nrf2 and target gene expression, while Wnt3a knockdown exacerbates CSE-induced Nrf2 suppression. Notably, Nrf2 deficiency does not affect the Wnt3a/β-catenin pathway, indicating unidirectional regulation from Wnt to Nrf2 [48]. Additionally, the antioxidant protein DJ-1 stabilizes Nrf2 by activating Wnt3a/β-catenin signaling, resulting in Nrf2-mediated upregulation of multidrug resistance-associated protein 1 (MRP1) and HO-1, thereby mitigating oxidative stress. Conversely, DJ-1 knockdown exacerbates inflammation induced by CSE and LPS, further highlighting its critical role in maintaining Nrf2 activity [27].

AMP-activated protein kinase (AMPK), a key cellular energy sensor, mitigates lung inflammation and oxidative stress in COPD. CS exposure suppresses AMPK phosphorylation, contributing to reduced Nrf2 expression [48,49]. AMPK activation by metformin increases Wnt3a, β-catenin, and phosphorylated Nrf2 levels, reducing pro-inflammatory cytokines in CSE-exposed NHBE cells. Importantly, this anti-inflammatory effect is abolished in Nrf2-deficient cells, highlighting Nrf2's central role in AMPK-mediated protection [48]. Furthermore, triterpene acids from loquat leaf restore AMPK phosphorylation and Nrf2 signaling in *C*S-exposed mice, alleviating lung injury and inflammation. This suggests that AMPK activation enhances Nrf2-mediated antioxidant responses, countering *C*S-induced oxidative stress [49].

Targeting the PI3K/Akt-Nrf2 axis offers therapeutic promise for COPD. CS exposure significantly downregulates PI3K expression, along with other upstream kinases such as PKC and mitogen-activated protein kinase (MAPK), impairing Nrf2 activation and antioxidant genes transcription, thereby exacerbating oxidative stress and inflammation [50,51]. The Bu-Shen-Fang-Chuan formula mitigates *C*S-induced pulmonary inflammation in rats by enhancing PI3K/Akt-mediated Nrf2 activation. This results in increased Akt phosphorylation, Nrf2 nuclear translocation, and antioxidant gene transcription, effectively counteracting oxidative damage and reducing inflammatory cytokines like TNF-α and IL-6 [51]. However, Ding-Chuan-Tang, another traditional Chinese medicine, inhibits PI3K/Akt signaling but paradoxically enhances Nrf2 stability and nuclear accumulation. This suggests that Nrf2 activation may occur via alternative pathways, such as MAPK or PKC, independent of PI3K/Akt signaling [26]. Therefore, despite inhibiting PI3K/Akt, Ding-Chuan-Tang's anti-inflammatory and antioxidant effects in COPD are partially mediated through Nrf2 upregulation, further underscoring the therapeutic potential of targeting Nrf2 activation for COPD management.

The MAPK family, including ERK, JNK, and p38 MAPK, plays a pivotal role in modulating Nrf2 activity, thereby influencing oxidative stress and inflammation in COPD [25,26,50,52]. CS exposure induces ERK phosphorylation (*p*-ERK), which phosphorylates Nrf2, promoting its nuclear translocation and binding to AREs to upregulate antioxidant genes [25,52]. While ERK-mediated Nrf2 activation offers protection against oxidative damage, excessive MAPK signaling can exacerbate inflammation and apoptosis. Inhibition of ERK, either pharmacologically or using the Recuperating lung decoction, reduces *p*-ERK levels, suppresses Nrf2 overexpression, and alleviates oxidative damage and inflammation in COPD models [52]. Furthermore, CS exposure activates JNK and p38 MAPK, contributing to increased apoptosis and inflammation in bronchial epithelial cells (BECs). Although less directly associated with Nrf2 activation than ERK, inhibition of JNK/p38 by the total alkaloid extract from Bulbus *Fritillariae Pallidiflorae* (BFP-TA) reduces oxidative stress and restores antioxidant capacity, suggesting potential crosstalk with Nrf2-mediated defenses [25].

NADPH oxidase (NOX) 4, a major ROS-generating enzyme upregulated in COPD, plays a crucial role in disrupting redox balance and impairing Nrf2 activity. Heavy metals, such as cadmium, lead, mercury, and chromium, elevate NOX4 expression in monocytes, impairing the Nrf2/GCLC/glutathione *S*-transferase (GST) antioxidant pathway. This disruption reduces GSH levels, exacerbating oxidative stress and worsening lung function in COPD patients [12]. Additionally, exposure to PM_2.5_ exacerbates COPD by inducing NOX4-mediated ROS overproduction, which further suppresses Nrf2 activity. This imbalance leads to mitochondrial dysfunction, excessive mitophagy, and inflammation [53]. Restoring the NOX4/Nrf2 equilibrium, either through NOX4 inhibition or antioxidant treatment, mitigates these effects, highlighting that targeting this redox imbalance represents a promising therapeutic strategy for COPD.

Sirtuin (SIRT)1, a NAD^+^-dependent deacetylase, is a critical regulator of Nrf2 activity in COPD. Astaxanthin alleviates *C*S-induced oxidative stress and inflammation by directly binding to and activating SIRT1. This activation enhances SIRT1's deacetylase function, resulting in Nrf2 deacetylation, stabilization, and nuclear translocation. Notably, SIRT1 inhibition abolishes the protective effects of astaxanthin, confirming the essential role of the SIRT1/Nrf2 axis [54]. Similarly, BFP-TA attenuates *C*S-induced oxidative stress in BEAS-2B cells by upregulating SIRT1, which activates Nrf2 and downstream antioxidant factors [25]. These findings collectively establish SIRT1 as a critical upstream activator of Nrf2, positioning it as a promising therapeutic target in COPD management.

Beyond well-established regulators above, emerging evidence highlights additional upstream factors modulating Nrf2 activity in COPD. These include dachshund homolog 1, damage-associated molecular patterns (DAMPs) signaling, pyruvate kinase M2 (Pkm2), cytochrome P450 family 2 subfamily A member 13 (CYP2A13), and metabolism-related gene thioredoxin reductase 1 (TXNRD1) [[55], [56], [57], [58], [59]]. These findings underscore the complexity of Nrf2 regulation in COPD, involving metabolic enzymes, xenobiotic metabolizers, danger signals, and transcriptional regulators, offering diverse potential therapeutic targets.

Collectively, the regulatory network governing Nrf2 in COPD reveals a disease-specific pattern of dysfunction that complements and reinforces the direct impairment of Nrf2 itself. The upstream pathways, while shared with other processes, are co-opted and dysregulated within the chronic, low-grade oxidative stress milieu of the COPD lung. For instance, CS suppresses protective pathways such as Wnt/β-catenin and AMPK, while it aberrantly activates stress-responsive kinases including p38 MAPK, thereby collectively contributing to Nrf2 instability and insufficient activation [25,27,49]. This represents a fundamental difference from other pathological conditions where these same pathways may serve to potentiate Nrf2 activity. The convergence of these dysregulated upstream signals, encompassing energy sensing through AMPK, redox balance mediated by NOX4 and SIRT1, and developmental signaling via Wnt, upon a compromised Nrf2 node underscores the centrality of its failure in COPD pathogenesis [27,49,53,54]. This intricate crosstalk, dysregulated by the specific etiology of COPD, ultimately defines the insufficient antioxidant and anti-inflammatory response that is a hallmark of the disease.

## 3 Functional roles and downstream effectors of Nrf2 in COPD pathogenesis

Nrf2 activation orchestrates a complex transcriptional program that mitigates COPD pathogenesis primarily by regulating key downstream effectors involved in antioxidant defense, inflammation resolution, and cellular homeostasis, as partially illustrated in Table 1 and further detailed in the following sections regarding downstream targets.

### 3.1 Nrf2-mediated regulation of antioxidant factors in COPD

Nrf2 activation combats oxidative stress in COPD primarily by transcriptionally upregulating key antioxidant enzymes and cytoprotective proteins.

HO-1, a critical enzyme in heme degradation, exerts antioxidant and anti-inflammatory effects through the production of bilirubin, carbon monoxide, and ferritin. As a primary Nrf2 target, HO-1 is central to cytoprotection. In COPD models, CS exposure inactivates Nrf2 and suppresses HO-1, exacerbating oxidative damage [60]. Conversely, Nrf2 activation, demonstrated by the activator RTA-408, upregulates HO-1 in PM_2.5_-exposed human BECs and rat models, mitigating oxidative stress and inflammation [61]. Multiple compounds and formulations protect against COPD by activating the Nrf2/HO-1 antioxidant pathway. Platycodin D, alantolactone, astaxanthin, and aucubin significantly mitigate *C*S-induced lung inflammation and oxidative damage in vivo and in vitro. Their protective effects, including enhanced antioxidant capacity and reduced pro-inflammatory cytokines, are mechanistically linked to promoted Nrf2 nuclear translocation and subsequent HO-1 upregulation [28,54,62,63]. Critically, Nrf2 deficiency or pharmacological inhibition abolishes this protection and worsens COPD pathology, underscoring the crucial role of the Nrf2/HO-1 axis [28]. Similarly, the efficacy of modified Jinshui Liujian decoction combined with Bajitian pill in COPD rats correlates with elevated Nrf2/HO-1 levels and reduced oxidative markers like malondialdehyde (MDA) [64]. The Nrf2/HO-1 pathway also modulates detoxification and glucocorticoid sensitivity [65,66]. CS exposure impairs MRP1 function, a transporter critical for effluxing toxic metabolites such as GSH conjugates of 4-hydroxynonenal (4-HNE), leading to cytotoxic accumulation and inflammation. Metformin restores MRP1 via Nrf2/HO-1, rescuing histone deacetylase 2 (HDAC2) activity and reducing glucocorticoid resistance in COPD models [66]. Non-pharmacological strategies also target this axis. Dental pulp stem cells transplantation elevates HO-1, improving lung function and reducing oxidative stress in elastase-induced COPD mice [67]. Treadmill exercise increases serum irisin, enhancing Nrf2/HO-1 expression and attenuating emphysema and neutrophil infiltration in *C*S-exposed mice [68]. Moreover, Nrf2 activation reduces apoptosis and pulmonary remodeling in COPD. Aucubin reduces lung cell apoptosis, fibrosis, and emphysema through Nrf2/HO-1 [28]. Similarly, auraptene improves respiratory function by restoring *E*-cadherin and reducing extracellular matrix deposition, effects that are mediated via Nrf2-driven regulation of oxidative and inflammatory mediators [5]. Collectively, the Nrf2/HO-1 axis is a pivotal therapeutic target for mitigating multiple aspects of COPD pathology.

NQO1, another critical Nrf2-regulated enzyme, combats oxidative stress and inflammation by detoxifying reactive quinones in COPD. Multiple natural agents, including allyl isothiocyanate, YPL-001, cryptotanshinone, and phycocyanin, mitigate COPD-associated oxidative stress and inflammation through a shared mechanism involving the activation of the Nrf2/NQO1 pathway. These compounds promote Nrf2 nuclear translocation, leading to the coordinated upregulation of HO-1 and NQO1. This enhanced Nrf2-driven antioxidant response has been shown to effectively counteract CSE-induced cellular damage in vitro and ameliorate COPD pathology in vivo, resulting in reduced oxidative injury, diminished inflammation, and improved lung function [3,[69], [70], [71]]. In contrast, quercitrin inhibits *C*S-induced oxidative damage in human BECs by suppressing Nrf2 nuclear translocation and downregulating HO-1 and NQO1 expression [72]. Despite some discrepancies, these studies emphasize that activating the Nrf2/NQO1 axis holds significant promise for alleviating COPD symptoms and slowing disease progression.

GCLC is a rate-limiting enzyme in GSH synthesis. Nrf2 directly regulates GCLC transcription, ensuring sufficient GSH levels to neutralize ROS. CS exposure depletes GSH levels and suppresses GCLC activity, thereby exacerbating oxidative damage in COPD. Furthermore, heavy metals such as cadmium and lead impair the Nrf2/GCLC/GSH axis in monocytes, leading to reduced plasma GSH levels and GST activity, contributing to worsened lung function in COPD patients [12]. Bioactive compounds, including Crocin, ginseng saponin Rb1, and azithromycin, restore GCLC expression and GSH levels in *C*S-exposed BECs and animal models via Nrf2 activation, countering oxidative stress and improving barrier function [50,73,74]. These findings highlight the Nrf2/GCLC/GSH pathway as a critical therapeutic target for counteracting oxidative stress and maintaining redox balance in COPD.

Nrf2 enhances the expression and activity of superoxide dismutase (SOD), catalase (CAT), GPX, and malic enzyme 1 (ME1). SOD and CAT are essential antioxidant enzymes that neutralize superoxide radicals and hydrogen peroxide, respectively, thereby protecting cells from oxidative damage. Nrf2 activation increases their expression, bolstering cellular antioxidant defenses. CSE exposure significantly reduces SOD and CAT activities in lung tissues, but co-treatment with crocin restores these enzymes through Nrf2 activation [50]. Similarly, astaxanthin upregulates SOD and CAT levels in CSE-treated BECs, further emphasizing the role of Nrf2 in enhancing antioxidant responses [54]. GPX reduces lipid hydroperoxides and hydrogen peroxide, preventing lipid peroxidation and cellular damage. Nrf2 deficiency or dysfunction impairs GPX activity, exacerbating oxidative stress. Crocin treatment improves GPX activity and mitigates CSE-induced lipid peroxidation through Nrf2 activation, suggesting its therapeutic potential in COPD management [50]. Additionally, Seeds of *Ginkgo biloba* L. reverses *C*S-induced reductions in SOD and GPX activities in COPD rats, while also decreasing MDA levels [75]. ME1, a NADPH-generating enzyme, is another target of Nrf2. Activation of Nrf2 rescues ME1 expression and reprograms metabolism, thereby improving cellular energetics and redox balance to restore macrophage function in COPD [2]. These findings underscore the therapeutic potential of Nrf2-mediated upregulation of antioxidant enzymes.

### 3.2 Nrf2-mediated inhibition of NF-κB in COPD

Dysregulation of nuclear factor-kappa B (NF-κB) drives inflammation in COPD. Nrf2 activation mitigates this by suppressing aberrant NF-κB signaling, thereby reducing both inflammation and oxidative damage.

Pharmacological activation of Nrf2 consistently demonstrates potent anti-inflammatory effects in COPD models by targeting the NF-κB pathway. Nrf2 activators, including forsythiaside, isoliquiritigenin, *loranthus tanakae* extract, *trans*-4,4′-dihydroxystilbene, Codonopsis Radix, YPL-001, and ginsenoside Rb1, mitigate inflammation through multiple mechanisms [29,70,73,[76], [77], [78], [79]]. These compounds suppress NF-κB activation, as evidenced by reduced phosphorylation of p65, diminished degradation of inhibitor of kappa B alpha (IκBα), and decreased nuclear translocation. This leads to the downregulation of key pro-inflammatory cytokines such as TNF-α and IL-1β, and a reduction in inflammatory cell infiltration, particularly neutrophils.

In addition to direct NF-κB inhibition, Nrf2 activation exerts antioxidative effects that indirectly suppress NF-κB signaling. Nrf2 activators, such as alantolactone, counteract oxidative stress, a potent NF-κB trigger, by upregulating antioxidant enzymes like HO-1, GPX, and SOD, and by reducing ROS and lipid peroxidation [63]. Furthermore, Nrf2 activation mitigates mitochondrial dysfunction, a hallmark of COPD that exacerbates inflammation through NF-κB pathways. By preserving mitochondrial integrity and reducing mitochondrial ROS production, Nrf2 activation further dampens NF-κB-driven inflammation. Studies show that Nrf2 deficiency exacerbates mitochondrial damage and inflammation, while activation restores mitochondrial function and effectively suppresses NF-κB signaling [80].

### 3.3 Nrf2-mediated regulation of NLRP3 inflammasome in COPD

Nrf2 activation plays a crucial role in mitigating inflammation in COPD, partly by regulating the NLRP3 inflammasome and pyroptosis, a pro-inflammatory cell death pathway. In *C*S-induced models, Nrf2 deficiency results in the upregulation of NLRP3, activation of caspase-1, and cleavage of gasdermin D (GSDMD) into its active form (GSDMD-N), driving pyroptosis and exacerbating inflammation [60,81]. Conversely, pharmacological activation of Nrf2 using agents such as dimethyl fumarate, propofol, Schisandrin A, and Quanzhen Yiqi decoction inhibits NLRP3 inflammasome assembly, caspase-1 activation, GSDMD cleavage, and the release of pro-inflammatory cytokines [60,[81], [82], [83]]. Notably, overexpression of NLRP3 reverses the anti-inflammatory effects of Nrf2 activation, confirming the critical role of Nrf2 in regulating NLRP3-mediated pyroptosis [60]. Furthermore, Nrf2 activation upregulates antioxidant enzymes, such as HO-1 and NQO-1, enhancing the cellular defense against oxidative stress. Zinc supplementation in *C*S-exposed mice alleviates alveolar damage and reduces IL-1β, IL-6, and IL-18 levels through Nrf2 signaling. However, this protective effect is abolished by the Nrf2 inhibitor ML385, highlighting the critical involvement of Nrf2 in these processes [4]. These findings highlight the therapeutic potential of targeting the Nrf2/NLRP3/pyroptosis axis in managing COPD-related inflammation.

The functional outcome of Nrf2 activation in COPD is the induction of a cytoprotective program that specifically counteracts the cardinal pathological features of the disease. The downstream effectors of Nrf2, such as HO-1, NQO1, and GCLC, are not merely generic antioxidants; their coordinated upregulation is essential for mitigating *C*S-induced emphysematous destruction, maintaining airway epithelial integrity, and resolving chronic neutrophilic inflammation [61,71]. These processes are hallmarks that define COPD. A particularly unique aspect of Nrf2's role is its governance over cell death pathways. Beyond suppressing apoptosis and pyroptosis, its integrated control of redox and iron homeostasis positions Nrf2 as a master regulator of ferroptosis. This iron-dependent cell death is increasingly recognized as a key driver of alveolar epithelial damage in COPD, a distinction that sets it apart from other obstructive lung diseases such as asthma [84]. Consequently, the functional failure of Nrf2 in COPD creates a specific vulnerability to ferroptosis, which in turn accelerates disease progression through mechanisms that are not as prominent in other pulmonary conditions.

## 4 Crosstalk between Nrf2 and ferroptosis in COPD

### 4.1 Core mechanisms of ferroptosis and the regulatory role of Nrf2

**Fig. 3:**
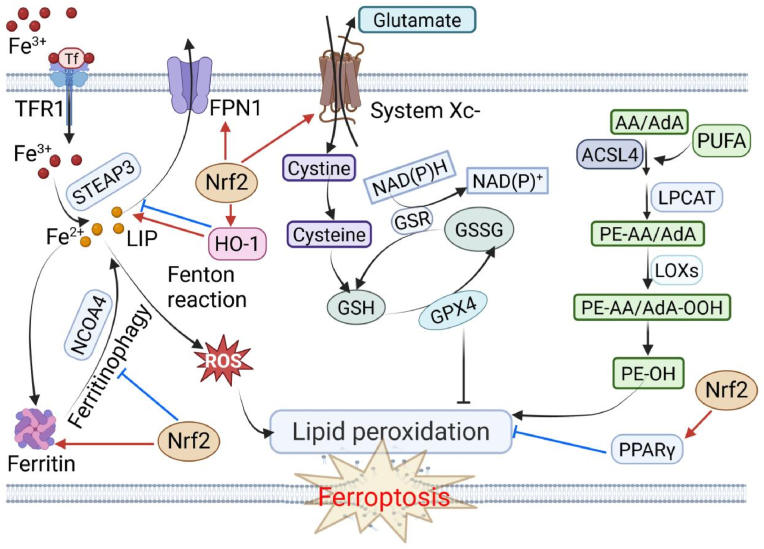
Mechanisms of ferroptosis and regulatory role of Nrf2. Ferroptosis is driven by three core processes: (1) Iron overload via dysregulated uptake (Tf/TFR1), storage (ferritin), release (ferritinophagy/NCOA4), and efflux (FPN1); (2) Collapse of the antioxidant system via impaired System Xc^−^ (SLC7A11)-mediated cystine uptake, GSH depletion, and GPX4 inactivation; and (3) Lipid peroxidation of PUFA-phospholipids, causing membrane damage. Nrf2 modulates ferroptosis at multiple levels: It promotes iron sequestration (FTH1) and export (FPN1) while inhibiting ferritinophagy to reduce labile iron; it activates System Xc^−^ (SLC7A11) and GPX4 to enhance antioxidant defense; it upregulates PPARγ to suppress lipid peroxidation and inflammation; and it induces HO-1, which exhibits dual roles depending on the cellular context.

Ferroptosis is an iron-dependent form of regulated cell death driven by the lethal accumulation of lipid peroxides. Its core mechanisms involve three interconnected pathological processes: iron overload, the collapse of GSH-dependent antioxidant system, and the peroxidation of polyunsaturated fatty acid (PUFA)-containing phospholipids [15,16,85]. The transcription factor Nrf2 serves as a master regulator of cellular redox homeostasis and exerts multi-faceted control over these ferroptotic processes (Fig. 3).

Dysregulated iron homeostasis is a key driver of ferroptosis. Cellular iron uptake is mediated by the transferrin (Tf)/transferrin receptor 1 (TFR1) complex [86]. Intracellular iron is stored in ferritin, while nuclear receptor coactivator 4 (NCOA4)-mediated ferritinophagy degrades ferritin to release iron back into the labile iron pool (LIP) [87]. Iron efflux is regulated by ferroportin 1 (FPN1) [88]. Dysregulation of this system leads to iron overload [89,90]. Surplus Fe^2+^ catalyzes the Fenton reaction, generating hydroxyl radicals that initiate lipid peroxidation, a central event in ferroptosis [16]. The System Xc^−^/GSH/GPX4 axis is a crucial antioxidant defense. System Xc^−^, composed of SLC7A11 and SLC3A2, imports cystine for GSH synthesis [91,92]. GSH serves as the essential cofactor for GPX4, which reduces toxic phospholipid hydroperoxides (PLOOHs) to harmless phospholipid alcohols (PLOHs), thereby preventing membrane lipid peroxidation [93,94]. Inhibition of this pathway, through SLC7A11 suppression, GSH depletion, or GPX4 inactivation, leads to lethal lipid peroxide accumulation and triggers ferroptosis [15,95,96]. Lipid peroxidation is the oxidative degradation of PUFAs within membrane phospholipids, constituting a hallmark of ferroptosis. This process is catalyzed by ROS and enzymes like lipoxygenases (LOXs) [97]. Acyl-CoA synthetase long-chain family member 4 (ACSL4) activates PUFAs and promotes their incorporation into membrane phospholipids, making them substrates for peroxidation [85,98]. The resulting lipid peroxides compromise membrane integrity and fluidity, leading to membrane rupture and cell death.

Nrf2 is a pivotal transcriptional regulator that modulates multiple aspects of ferroptosis susceptibility. It governs cellular defenses against ferroptosis by controlling iron homeostasis, the System Xc^−^/GSH/GPX4 axis, lipid peroxidation, and through crosstalk with other pathways such as PPARγ [15,19]. Consequently, restoring Nrf2 activity presents a promising therapeutic strategy to mitigate oxidative stress and prevent ferroptosis. Specifically, Nrf2 directly transactivates genes that maintain iron homeostasis, including ferritin heavy chain 1 (FTH1) and FPN1, thereby promoting iron storage and export to reduce the LIP and limit Fenton reaction-derived ROS [99,100]. Nrf2 activation also suppresses ferritinophagy, preventing iron overload and lipid peroxidation. Studies have demonstrated that Nrf2 activators alleviate iron overload-induced damage by enhancing iron storage and efflux, underscoring its role in maintaining iron homeostasis and counteracting ferroptosis [101,102]. Furthermore, Nrf2 transcriptionally activates SLC7A11, promoting cystine uptake for GSH synthesis and GPX4 activity, thus protecting cells from oxidative damage and ferroptosis [15]. Nrf2 also regulates lipid peroxidation through its target gene HO-1, which exerts a dual, context-dependent role. Moderate HO-1 induction alleviates oxidative stress through biliverdin production. However, excessive HO-1 activity, particularly under conditions of iron dysregulation, can exacerbate ferroptosis by increasing intracellular free iron levels [103,104]. Therefore, the overall effect of HO-1 depends on the balance between its cytoprotective and pro-ferroptotic functions. Additionally, Nrf2 interacts with PPARγ, enhancing its expression through binding to its promoter region. PPARγ, in turn, inhibits lipid peroxidation and inflammation, further suppressing ferroptosis [102]. Thus, Nrf2 stands as a central guardian against ferroptosis, and its dysfunction is intimately linked to the pathogenesis of COPD.

### 4.2 Dysregulation of the Nrf2-ferroptosis axis in COPD pathogenesis

In COPD, the protective crosstalk between Nrf2 and ferroptosis is fundamentally disrupted. A hallmark of the disease is the pervasive dysfunction of the Nrf2 pathway, which creates a permissive environment for ferroptosis execution. The iron overload, GPX4 suppression, and elevated lipid peroxidation observed in COPD can therefore be viewed as direct consequences of a failing Nrf2-ferroptosis defense axis [15,101,102]. This dysfunction is driven by key triggers like CS and environmental pollutants, and is cemented through epigenetic mechanisms.

#### 4.2.1 Nrf2 dysfunction: the permissive foundation for ferroptosis

Analysis of lung tissue from COPD patients consistently reveals impaired Nrf2 signaling. This occurs through multiple mechanisms, including Keap1-mediated ubiquitination and proteasomal degradation, as well as promoter hypermethylation that suppresses Nrf2 transcription [23,105]. This Nrf2 impairment diminishes the expression of cytoprotective genes, weakening cellular defenses against oxidative stress and creating a permissive environment for ferroptosis initiation and propagation. Analysis of COPD and ferroptosis-related datasets has identified a cluster of Nrf2-dependent ferroptosis-related hub genes, including NQO1, GPX2, SLC7A11, TXNRD1, and SRXN1 [106]. While these genes may be upregulated adaptively in the early stages, Nrf2 deficiency becomes predominant in advanced disease, correlating with disease severity and promoting ferroptosis [19,23,106,107]. Gene set enrichment analysis further confirms the pivotal, though dysregulated, role of Nrf2 in COPD progression. Consequently, the failure of Nrf2-mediated antioxidant defense system directly exacerbates lipid peroxidation, iron dysregulation, and ferroptosis in respiratory epithelial cells, thereby contributing to emphysema, airway inflammation, and disease progression. Nrf2 knockout mice exhibit heightened susceptibility to *C*S-induced lung damage, with amplified ferroptosis markers [19,106].

#### 4.2.2 Key triggers and the resultant Nrf2 dysfunction

CS is a central trigger that disrupts iron homeostasis by upregulating TFR1 and activating NCOA4-mediated ferritinophagy, leading to iron overload [108,109]. Crucially, CS impairs the Nrf2-GPX4 axis by promoting the degradation of milk fat globule epidermal growth factor 8 (MFG-E8), a stabilizer of GPX4 and SLC7A11, and by downregulating GCLC [110,111]. Environmental pollutants like diesel exhaust particles (DEPs) and microplastics exacerbate this situation. Polystyrene microplastics (*P*S-MPs) accumulate in lungs of COPD patients, exacerbating mitochondrial dysfunction and iron overload. *P*S-MPs induce ferroptosis through mitochondrial ROS-mediated autophagy, characterized by elevated ACSL4, reduced GPX4, and ferritinophagy-driven iron release [112]. Similarly, DEPs disrupt mitochondrial redox balance, increase lipid peroxidation, and inhibit antioxidant defenses such as GPX4 and GCLC. The exposure to DEPs leads to excessive ROS production through mitochondrial electron transport chain complex upregulation, and concurrently downregulates the Hippo pathway effector TAZ, a GPX4 transcriptional regulator, which further exacerbates oxidative damage [111]. These insults collectively suppress Nrf2 activity and its target genes, creating a vicious cycle where ferroptosis drivers are amplified while defensive pathways are weakened.

#### 4.2.3 Epigenetic silencing of the Nrf2 pathway

Epigenetic mechanisms play a critical role in the sustained suppression of the Nrf2-ferroptosis defense axis in COPD, even after smoking cessation. Hypermethylation of the Nrf2 promoter is a prominent alteration that suppresses its expression, thereby impairing the entire downstream antioxidant network [23]. Furthermore, active DNA demethylation of the GPX4 promoter by the TET2 dioxygenase is essential for GPX4 transcription. In COPD, TET2 downregulation leads to GPX4 promoter hypermethylation and suppressed expression, sensitizing cells to ferroptosis [113]. Post-transcriptionally, the m6A demethylase FTO exacerbates *C*S-induced ferroptosis in BECs by erasing m6A modifications, leading to the downregulation of *anti*-ferroptotic Nrf2 targets like SLC7A11 and NQO-1 [114]. Furthermore, CSE exposure upregulates the histone demethylase JMJD3, which removes repressive H3K27me3 marks at the ACSL4 promoter. This chromatin remodeling enhances ACSL4 transcription, facilitating PUFA incorporation into membrane phospholipids and increasing susceptibility to lipid peroxidation [109]. Knockdown of JMJD3 reduces ACSL4 expression, lipid peroxidation, and cell death in BECs. These epigenetic modifications provide a mechanistic basis for the persistent ferroptosis phenotype in COPD and highlight the therapeutic potential of targeting these pathways to restore Nrf2 function.

### 4.3 Therapeutic implications: restoring Nrf2 activity to suppress ferroptosis

The central role of Nrf2 dysfunction in promoting ferroptosis highlights its promise as a therapeutic target. Strategies to activate Nrf2 or mimic its action have shown significant efficacy in preclinical COPD models [15,84,115]. These interventions primarily function by restoring the core defensive axes outlined in Fig. 3 and summarized in Table 2.

**Table 2:** Core axes of the Nrf2-ferroptosis interplay and therapeutic interventions in COPD.

Core axis/mechanism	Key molecular targets	Effect on ferroptosis in COPD	Representative therapeutic agents
Nrf2/SLC7A11/GPX4 axis	SLC7A11, GCLC, GSH, GPX4	Core defense. Nrf2 activation enhances cystine uptake, GSH synthesis, and GPX4 activity, directly inhibiting lipid peroxidation.	Dihydroquercetin, Acacetin, *Thesium chinense* Turcz., Shenqi Tiaoshen formula, Sodium pyruvate
Nrf2/HO-1 axis	HO-1, Fe^2+^	Dual role. Protective in early stages via bilirubin/CO; can become pro-ferroptotic in advanced COPD due to iron release.	Scutellarein (inhibits excessive activation)
Nrf2/PPARγ/ferritinophagy axis	PPARγ, NCOA4, FTH1	Reduces labile iron pool. Nrf2-upregulated PPARγ suppresses NCOA4-mediated ferritinophagy, limiting Fe^2+^ release.	Hydrogen sulfide
Nrf2/lipid metabolism regulation	ACSL4, ALOX5, ALOX15	Suppresses lipid peroxidation. Nrf2 downregulates enzymes that incorporate (ACSL4) or oxidize (ALOXs) PUFAs into membranes.	*Thesium chinense* Turcz. (inhibits ACSL4, ALOX5), Scutellarein (inhibits ALOX15)
Nrf2/SIRT3/iNOS axis	SIRT3, iNOS	Improves mitochondrial function. Nrf2 transactivates SIRT3, which can suppress iNOS, reducing nitrosative stress and lipid peroxidation.	Ganoderic acid D (SIRT3 activator)
Nrf2/general antioxidant response	NQO1, TXNRD1, SRXN1, GST	Broad-spectrum antioxidant support. Upregulation of these enzymes reduces overall cellular oxidative stress, creating an environment resistant to ferroptosis.	Multiple Nrf2 activators

#### 4.3.1 The Nrf2/SLC7A11/GPX4 axis: a core defense against lipid peroxidation

The Nrf2/SLC7A11/GPX4 axis is central to Nrf2-mediated protection against ferroptosis in COPD. Nrf2 transactivates SLC7A11, crucial for cystine import and subsequent GSH synthesis [106]. GSH is a major cellular antioxidant. GPX4, a key Nrf2 target, utilizes GSH as a cofactor to detoxify LOOH into LOH, thereby preventing iron-catalyzed lipid peroxidation, a hallmark of ferroptosis [93,94]. CS exposure inhibits SLC7A11 and GPX4 expression in airway epithelial cells and macrophages, primarily through Nrf2 suppression [13,107,115,116]. This downregulation impairs cystine uptake, depletes GSH, inactivates GPX4, and promotes lipid peroxidation accumulation, ultimately leading to ferroptosis. Pharmacological activation of Nrf2 using dihydroquercetin, Thesium chinense Turcz., Shenqi Tiaoshen formula, acacetin, sulforaphane, or sodium pyruvate restores SLC7A11 and GPX4 expression, replenishes GSH, reduces lipid peroxidation, thereby protecting against *C*S-induced ferroptosis in vitro and in vivo models of COPD [15,84,85,[115], [116], [117]]. These protective effects are abolished by Nrf2 silencing or inhibition, underscoring the pivotal role of Nrf2 in this process [15,84,116]. Additionally, Nrf2 deficiency exacerbates ferroptosis in response to PM_2.5_ or CS [19,118]. Single-cell analyses further reveal that dysregulation of this axis in macrophages correlates with COPD progression [13].

#### 4.3.2 The Nrf2/HO-1 axis: a double-edged sword in ferroptosis regulation

HO-1, a key Nrf2 target, exhibits a context-dependent and dual role in regulating ferroptosis during COPD pathogenesis, effectively functioning as a double-edged sword. The shift of HO-1 from a cytoprotective molecule to a pro-ferroptotic driver is primarily determined by three interconnected factors: the severity and duration of oxidative stress, the cellular capacity to manage liberated iron, and the overall integrity of parallel antioxidant defense systems, with the GPX4 axis being particularly critical [24,105,106].

Under conditions of moderate or acute oxidative stress, which are characterized by transient Nrf2 activation, intact GPX4 expression and activity, and functional cellular iron-sequestration mechanisms such as ferritin, the induction of HO-1 via the Nrf2 pathway is predominantly protective. In this scenario, the catalytic products of HO-1, namely the potent antioxidant bilirubin and the anti-inflammatory cytoprotective molecule carbon monoxide, effectively counteract oxidative stress and inflammation, thereby mitigating the initiation of ferroptosis [106].

The transition to a detrimental role occurs under the sustained and severe oxidative stress that characterizes advanced COPD. This pathological state is defined by a triad of key impairments including chronic Nrf2 dysfunction, which has been observed in myofibroblasts from COPD patients and contributes to disease progression, significant GPX4 suppression, and pre-existing iron dyshomeostasis typified by an elevated labile iron pool [119]. In this compromised environment, persistent HO-1 activation becomes a primary driver of ferroptosis. The critical mechanism underlying this pathological switch is the relentless release of ferrous iron via HO-1-mediated heme degradation. When this iron is released into a cell where the antioxidant capacity is already compromised due to GSH depletion and GPX4 inactivation, and where iron storage and export systems are overwhelmed, it exacerbates iron overload. The resultant amplification of Fenton reactions dramatically elevates lipid peroxidation, directly overwhelming cellular defenses and executing ferroptosis [14,105]. Mechanistically, in COPD models, CSE induces a self-amplifying loop of HO-1 upregulation via NOX-derived ROS and Nrf2-dependent transcription in BECs, as demonstrated in studies of phytochemical interventions [24]. The pivotal role of HO-1 in driving ferroptosis under these conditions is evidenced by the reversal of cell death upon pharmacological inhibition with agents such as ZnPP and upon genetic knockdown of HO-1. Correspondingly, in vivo, CSE-induced lung damage and elevated ferroptosis markers are significantly rescued by HO-1 inhibition or treatment with the ferroptosis inhibitor Ferrostatin-1 [105].

In summary, the net effect of HO-1 in COPD is not static but evolves with disease progression. Its functional transition hinges on the integrity of the cellular context, particularly the GPX4 system and iron-handling capacity. While Nrf2-mediated HO-1 induction offers initial protection, its sustained activity within a degraded redox and iron-handling environment paradoxically fuels the ferroptotic process. This delineation underscores the therapeutic imperative for context-dependent HO-1 modulation. For instance, the flavonoid scutellarein demonstrates this principle by blocking excessive Nrf2 and HO-1 activation to reduce HO-1 and ferrous iron-driven ferroptosis, proving effective in experimental models [14].

#### 4.3.3 Additional Nrf2-mediated protective mechanisms against ferroptosis

Beyond the core SLC7A11/GPX4 axis and the context-dependent HO-1 pathway, Nrf2 regulates ferroptosis in COPD through several other interconnected mechanisms, including the Nrf2/PPARγ/ferritinophagy axis, the ROS/Nrf2/SIRT3/inducible nitric oxide synthase (iNOS) axis, and lipid metabolism [15,19,106,120].

Nrf2 activation upregulates PPARγ, which suppresses NCOA4-mediated ferritinophagy and stabilizes ferritin, reducing labile Fe^2+^ release. In COPD models, Nrf2 deficiency disrupts this axis, increasing ferritinophagy and promoting iron overload, lipid peroxidation, and ferroptosis. Protective agents like hydrogen sulfide have been shown to mitigate PM-induced injury by restoring the Nrf2/PPARγ/ferritinophagy pathway [19].

Furthermore, Nrf2 directly transactivates SIRT3, a mitochondrial deacetylase essential for maintaining mitochondrial function and redox balance. In COPD, *C*S-induced ROS suppress the Nrf2/SIRT3 axis, leading to increased iNOS expression and excessive nitric oxide production. This impairs mitochondrial function, enhances nitrosative stress, and, in combination with ROS, exacerbates lipid peroxidation and ferroptosis. Restoring Nrf2/SIRT3 signaling helps mitigate ferroptosis by enhancing antioxidant defenses and suppressing iNOS activity [120].

Nrf2 also regulates ferroptosis by modulating lipid metabolism proteins. Nrf2 activation downregulates pro-ferroptotic lipid metabolism proteins, including ACSL4, which promotes the incorporation of PUFAs into cell membranes, and lipoxygenases like ALOX5 and ALOX15, which directly catalyze PUFA peroxidation. By reducing the availability of these lipid substrates for peroxidation, Nrf2 helps inhibit ferroptosis [14,15].

Additionally, Nrf2 upregulates a wide range of antioxidant and detoxifying enzymes, including NQO1, TXNRD1, SRXN1, and GST [106]. While the direct links of these enzymes to ferroptosis inhibition may not be as clearly defined as those of SLC7A11 or GPX4, collectively they play a significant role in reducing cellular oxidative stress and ROS levels, creating an environment less conducive to ferroptosis.

### 4.4 Biomarker potential of the Nrf2-ferroptosis axis

The interplay between Nrf2 dysfunction and ferroptosis activation offers valuable biomarker potential for COPD prognostication and monitoring. For these biomarkers to achieve clinical utility, rigorous validation using standardized methodologies across independent patient cohorts and a clear link to disease progression are paramount.

Promising candidates include the ratio of soluble TFR1 (sTFR1) to GPX4, along with lipid peroxidation products such as MDA and 4-HNE [18,121]. The ratio of sTFR1 to GPX4 in serum reflects the balance between iron demand and antioxidant defense. This ratio increases with COPD severity and correlates with reduced exercise capacity and exacerbation risk, serving as a composite indicator of the systemic ferroptosis burden and impaired Nrf2 activity [18]. Lipid peroxidation products MDA and 4-HNE, which are ubiquitously elevated in COPD biofluids with levels negatively correlating with forced expiratory volume in 1 s (FEV_1_) and the FEV_1_/forced vital capacity (FVC) ratio, can be detected in bronchoalveolar lavage fluid, sputum, or serum [122]. While assays like the thiobarbituric acid reactive substances assay are commonly used, mass spectrometry-based lipidomics is emerging as the gold standard. This advanced technique allows for specific and sensitive quantification of oxidized phospholipid species like phosphatidylethanolamine-AA/AdA-OOH, thereby providing a more direct measure of ferroptotic activity [121]. Additionally, quantitative susceptibility mapping MRI offers a non-invasive method to assess tissue iron deposition, for example, in the hippocampus, linking pulmonary ferroptosis to systemic complications like cognitive decline [123].

Critically, these biomarkers demonstrate dynamic changes. Serum levels of sTFR1 and MDA are significantly elevated during acute exacerbations of COPD compared to stable disease. Monitoring these biomarkers could help identify patients at high risk for frequent exacerbations [23]. Integrating ferroptosis biomarkers with clinical data holds promise for refining COPD staging. A proposed model suggests that early-stage COPD may be characterized by a compensatory upregulation of antioxidant pathways like Nrf2, whereas advanced stages are marked by their collapse. This leads to a dominant ferroptosis signature typified by a high sTFR1 to GPX4 ratio and elevated lipid peroxides. This concept is supported by findings that Nrf2 pathway activity in peripheral blood mononuclear cells is systemically altered in COPD [20].

While preclinical findings are promising, translational challenges remain, including the optimization of lung-targeted therapies and the standardization of biomarker assays across multicenter cohorts. Nevertheless, the Nrf2-ferroptosis axis represents a viable precision medicine target for COPD. The ongoing validation of these biomarkers in large, prospective studies is the critical next step toward their definitive integration into clinical practice for patient stratification, prognosis, and therapeutic monitoring.

## 5 Conclusion and perspectives

**Fig. 4:**
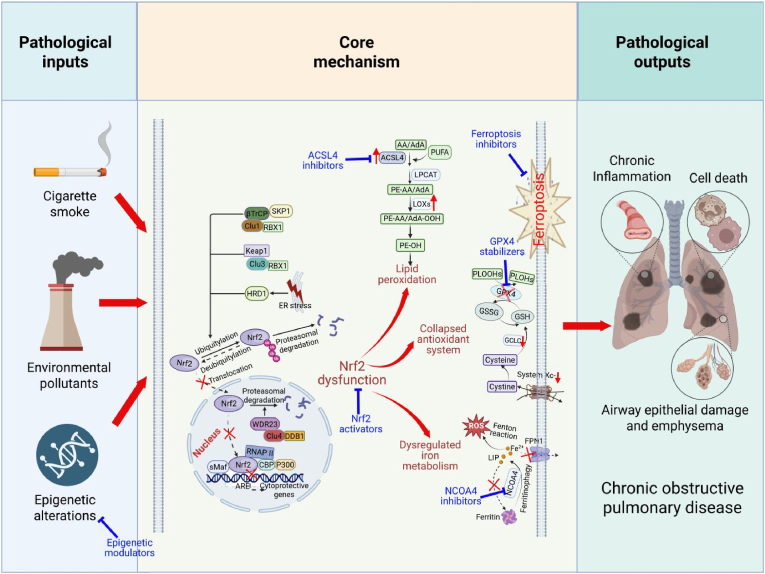
The core axis of Nrf2 dysfunction and ferroptosis in COPD pathogenesis and therapeutic interventions. This schematic summarizes the integrated regulatory network linking Nrf2 signaling to ferroptosis in the pathogenesis of COPD. (1) Pathological inputs: Chronic environmental insults, notably cigarette smoke and particulate matter, alongside epigenetic alterations such as promoter hypermethylation of Nfe2l2 and GPX4, are primary drivers of Nrf2 Dysfunction in COPD; (2) Core mechanism: Impaired Nrf2 signaling, resulting from Keap1-dependent degradation, GSK3β/βTrCP-mediated ubiquitination, etc., fails to sustain the expression of its cytoprotective target genes. This loss of transcriptional regulation de-represses three core pathways that execute ferroptosis, including dysregulated iron metabolism, antioxidant system collapse, and membrane lipid peroxidation; (3) Pathological outputs: The execution of ferroptosis directly contributes to hallmark COPD pathologies, including airway epithelial damage, emphysema, chronic inflammation, and cell death. Targeting this axis offers promising strategies, including Nrf2 activators, ferroptosis inhibitors, specific pathway inhibitors like ACSL4 inhibitors, GPX4 stabilizers, NCOA4 inhibitors, and epigenetic modulators.

This review summarizes the pivotal role of the Nrf2 signaling pathway in COPD pathogenesis and its critical regulatory interplay with ferroptosis. Nrf2 orchestrates a complex cytoprotective network, activated by diverse upstream signals such as Wnt/β-catenin, AMPK, PI3K/Akt, and SIRT1. Upon activation, Nrf2 transactivates several downstream effectors, including antioxidants, enzymes involved in GSH synthesis, detoxification and efflux proteins, and anti-inflammatory mediators. Nrf2 dysfunction is a core pathological feature in COPD, with consistent evidence from patients and models demonstrating impaired Nrf2 signaling. This disruption weakens the cellular antioxidant response, making the lungs more vulnerable to chronic oxidative stress and inflammation, key drivers of COPD pathology. Importantly, both pharmacological and genetic enhancement of Nrf2 signaling consistently ameliorate experimental COPD features. The intricate relationship between Nrf2 dysfunction and the execution of ferroptosis forms a core pathogenic axis in COPD, the overview of which is synthesized in Fig. 4.

Ferroptosis is a significant contributor to COPD pathogenesis, evidenced by hallmark features in diseased lungs, including iron overload, depletion of the GSH/GPX4 axis, and extensive lipid peroxidation. Environmental factors, such as CS, pollutants, and electronic nicotine delivery systems, along with epigenetic dysregulation, create a ferroptosis-permissive microenvironment. This microenvironment promotes epithelial barrier disruption, macrophage dysfunction, and exacerbates inflammation and tissue remodeling. Crucially, Nrf2 emerges as a master regulator of cellular resistance to ferroptosis. Its activation inhibits ferroptosis by enhancing the System Xc-/GSH/GPX4 axis, regulating iron metabolism, suppressing pro-ferroptotic lipid metabolism, modulating the context-dependent activity of HO-1, and upregulating broad-spectrum antioxidants like NQO1, TXNRD1, and SRXN1. In contrast, Nrf2 deficiency facilitates ferroptosis and exacerbates lung injury. Therapeutic restoration of Nrf2 activity effectively mitigates ferroptosis and improves lung function in experimental models.

Consequently, targeting the Nrf2-ferroptosis axis represents a highly promising therapeutic strategy for COPD. Several key research and development avenues offer significant potential, building on the mechanisms summarized in Table 1, Table 2. First, there is a critical need to develop potent, specific, and safe Nrf2 activators that target the various upstream pathways (Table 1) or core *anti*-ferroptotic axes (Table 2). This involves moving beyond traditional electrophilic Kelch modifiers, such as sulforaphane and bardoxolone methyl. In addition, natural compounds such as dihydroquercetin, scutellarein, and certain traditional Chinese medicine formulas have shown significant preclinical promise in activating Nrf2, offering potential therapeutic benefits. Another key strategy involves direct inhibition of ferroptosis using inhibitors like Ferrostatin-1, Liproxstatin-1, and iron chelators such as deferoxamine. Targeting specific pathway nodes, such as stabilizing GPX4, inhibiting ACSL4, and suppressing NCOA4, also offers potential therapeutic advantages in treating COPD. Additionally, combining Nrf2 activators with ferroptosis inhibitors or agents targeting other COPD pathologies may enhance therapeutic outcomes, and this approach warrants further exploration. Precision medicine is another promising approach, leveraging validated ferroptosis biomarkers to optimize patient stratification, prognosis, and therapeutic monitoring. The sTFR1/GPX4 ratio, lipid peroxidation products such as 4-HNE and MDA, and quantitative susceptibility mapping MRI for iron quantification could all play an important role in improving patient management. The development of advanced lung-targeted delivery systems, such as nanoparticles and inhalable formulations, is crucial for enhancing the efficacy of these therapeutic strategies while minimizing systemic side effects. In parallel, epigenetic modulation strategies, such as reversing Nrf2 promoter hypermethylation using DNMT inhibitors or regulating ferroptosis-associated epigenetic factors like TET2, FTO, and JMJD3, could provide additional avenues for novel therapeutic interventions in COPD. Lastly, advances in transcriptomics, particularly single-cell RNA sequencing and spatial transcriptomics, could offer critical insights into cell-type-specific dysregulation of the Nrf2 and ferroptosis pathways within the COPD lung microenvironment. This knowledge could guide the development of more precisely targeted and effective interventions for COPD patients.

Despite significant progress, several challenges remain in targeting the Nrf2-ferroptosis axis for COPD therapy. One of the key issues is the dual role of HO-1 in ferroptosis, which necessitates a precision medicine approach. Therapeutic strategies must carefully evaluate the disease stage and the specific redox-iron status of the lung microenvironment to determine whether to potentiate or inhibit the HO-1 pathway, as its beneficial effects are contingent upon a functional GPX4 system and controlled iron metabolism. Moreover, translating promising preclinical findings, particularly those involving natural compounds and novel ferroptosis inhibitors, into safe and effective clinical treatments necessitates rigorous clinical trials to ensure efficacy and safety. It is also crucial to define the precise contribution of ferroptosis relative to other cell death pathways, such as apoptosis and pyroptosis, across different COPD phenotypes and stages. Additionally, understanding the systemic impact of lung ferroptosis, particularly its effects on skeletal muscle atrophy and cognitive decline, will be critical for developing strategies to mitigate these broader consequences. Addressing these challenges is vital for harnessing the potential of the Nrf2-ferroptosis axis to develop transformative therapies for COPD, a disease that continues to lack effective disease-modifying treatments.

## Funding

This work was supported by the 10.13039/501100001809National Natural Science Foundation of China [grant number 82172551].

## CRediT authorship contribution statement

**Qian Gao:** Conceptualization, Investigation, Writing – original draft, Writing – review & editing. **Yayun Mao:** Conceptualization, Investigation, Writing – review & editing. **Shu Xie:** Conceptualization, Investigation, Writing – review & editing. **Dandan Liu:** Writing – review & editing. **Yifan Lv:** Writing – review & editing. **Xiaodan Liu:** Conceptualization, Supervision, Writing – review & editing. **Weibing wu:** Funding acquisition, Supervision, Writing – review & editing.

## Declaration of competing interest

The authors declare that they have no known competing financial interests or personal relationships that could have appeared to influence the work reported in this paper.

## Figures and Tables

**Table:** 

4-HNE	4-Hydroxynonenal
AA	Arachidonic acid
ACSL4	Acyl-CoA synthetase long-chain family member 4
AdA	Adrenic acid
AMPK	AMP-activated protein kinase
ARE	Antioxidant response element
BECs	Bronchial epithelial cells
BFP-TA	Total alkaloid extract from Bulbus Fritillariae Pallidiflorae
BTB	Broad complex, tramtrack, bric-a-brac
CAT	Catalase
CBP	CREB-binding protein
CNC-bZIP	Cap'n'Collar basic leucine zipper
COPD	Chronic obstructive pulmonary disease
CS	Cigarette smoke
CSE	Cigarette smoke extract
Cul	Cullin
CYP2A13	Cytochrome P450 family 2 subfamily A member 13
DAMPs	Damage-associated molecular patterns
DDB1	DNA damage-binding protein 1
DEPs	Diesel exhaust particles
ER	Endoplasmic reticulum
ERK	Extracellular signal-regulated kinases
FEV_1_	Forced expiratory volume in 1 s
FPN1	Ferroportin 1
FSP1	Ferroptosis suppressor protein 1
FTH1	Ferritin heavy chain 1
FVC	Forced vital capacity
GCLC	Glutamate-cysteine ligase catalytic subunit
GPX	Glutathione peroxidase
GSDMD	Gasdermin D
GSH	Glutathione
GST	Glutathione *S*-transferase
HDAC2	Histone deacetylase 2
HO-1	Heme oxygenase-1
HRD1	HMG-CoA reductase degradation protein 1 (SYVN1)
IκBα	Inhibitor of kappa B alpha
iNOS	Inducible nitric oxide synthase
IREB2	Iron-responsive element-binding protein 2
IVR	Intervening region
JMJD3	Jumonji domain-containing protein 3
JNK	*c*-Jun *N*-terminal kinase
Keap1	Kelch-like ECH-associated protein 1
LIP	Labile iron pool
LOOH	Lipid hydroperoxides
LOX	Lipoxygenase
MAPK	Mitogen-activated protein kinase
MDA	Malondialdehyde
ME1	Malic enzyme 1
MFG-E8	Milk fat globule epidermal growth factor 8
MMP	Matrix metalloproteinase
MRP1	Multidrug resistance-associated protein 1
NCOA4	Nuclear receptor coactivator 4
Neh	Nrf2-ECH homology
NF-κB	Nuclear factor-kappa B
NHBE	Normal human bronchial epithelial
NOX	NADPH oxidase
NQO1	NAD(P)H quinone oxidoreductase 1
Nrf2	Nuclear factor erythroid 2-related factor 2
PE	Phosphatidylethanolamine
PERK	protein kinase R (PKR)-like endoplasmic reticulum kinase
PKC	Protein kinase C
Pkm2	Pyruvate kinase M2
PLOH	Phospholipid alcohol
PLOOH	Phospholipid hydroperoxide
PPARγ	Peroxisome proliferator-activated receptor gamma
PRDX6	Peroxiredoxin 6
*P*S-MPs	Polystyrene microplastics
PTGS2	Prostaglandin-endoperoxide synthase 2 (COX2)
PUFA	Polyunsaturated fatty acid
RBX	RING-box protein
ROS	Reactive oxygen species
RXRα	Retinoid X receptor alpha
sTFR1	Soluble transferrin receptor 1
SIRT	Sirtuin
SKP1	*S*-phase kinase-associated protein-1
sMaf	Small Maf proteins
SOD	Superoxide dismutase
STEAP3	Six-transmembrane epithelial antigen of prostate 3
Tf	Transferrin
TFR1	Transferrin receptor 1
TXNRD1	Thioredoxin reductase 1
βTrCP	β-transducing repeat-containing protein

## Data Availability

No data was used for the research described in the article.
